# Epstein–Barr virus receptors: classification, functional mechanisms, and pathogenic implications

**DOI:** 10.1128/jvi.00291-26

**Published:** 2026-05-11

**Authors:** Hezirui Gu, Shuran Yang, Liting Song, Xiaolu Wang, Xiang Zheng, Jian Ma

**Affiliations:** 1 Hunan Key Laboratory of Cancer Metabolism Changsha, Hunan Cancer Hospital/The Affiliated Cancer Hospital of Xiangya School of Medicine, Central South University, Changsha, Hunan, China; 2 Xiangya School of Basic Medicine, Cancer Research Institute, Central South University 841896 , Changsha, Hunan, China; 3 Key Laboratory of Carcinogenesis and Cancer Invasion of the Chinese Ministry of Education, NHC Key Laboratory of Carcinogenesis, Hunan Key Laboratory of Nonresolving Inflammation and Cancer, Changsha, Hunan, China; 4 Department of Pathology, The First Affiliated Hospital of Guilin Medical University, Guilin, Guangxi, China; 5 Furong Laboratory 725122 , Changsha, Hunan, China; Universiteit Gent, Merelbeke, Belgium

**Keywords:** Epstein-Barr virus, EBV receptors, CD21, EphA2, R9AP, DSC2

## Abstract

Epstein-Barr virus (EBV) is a widely prevalent γ-herpesvirus that primarily infects human B cells and epithelial cells. It is closely associated with the development of various lymphomas and epithelial cancers, posing a significant threat to human life and health. The infection process of EBV relies on interactions between the virus and specific receptors on the surface of host cells, including CD21, NRP1, EphA2, and, more recently, discovered receptors such as R9AP and DSC2. Currently, no specific drugs targeting EBV have been developed, making further research on EBV receptors particularly crucial. This review summarizes the classification, structural characteristics, and molecular mechanisms of the EBV receptors identified to date and compares the differential pathways through which these receptors function in B cells and epithelial cells. Furthermore, this review discusses the potential applications of targeting EBV receptors in antiviral therapy, vaccine development, and cancer immunotherapy, while addressing the limitations of current research and future directions. The aim is to broaden the scope of future research in the field of EBV receptors and provide new perspectives for the prevention and treatment of EBV-associated diseases.

## INTRODUCTION

Epstein-Barr virus (EBV) is a widely disseminated γ-herpesvirus, infecting approximately 90% of adults worldwide (1). It is the first virus definitively linked to the development of human tumors. Since its first isolation from a Burkitt lymphoma patient in Africa in 1964 (2), EBV has become a major subject of research in virology, oncology, and immunology due to its complex infection mechanisms and close associations with various diseases.

In the context of tumors, EBV is closely associated with the development of various human cancers, among which nasopharyngeal carcinoma represents an important cancer type (3). Additionally, hematologic malignancies such as Hodgkin lymphoma (4), Burkitt lymphoma (5), NK/T-cell lymphoma (6), and gastric carcinoma (7) have also been confirmed to be closely associated with EBV. EBV is also a driving factor in the development of post-transplant lymphomas (8). It is estimated that in 2020, the number of new cancer cases globally caused by EBV ranged from 239,700 to 357,900, with cancer-related deaths between 137,900 and 208,700. The cancer burden attributable to EBV accounted for approximately 1.3%–1.9% of the global total burden (8). In addition, EBV is increasingly recognized as a potential contributor to the pathogenesis of certain autoimmune diseases, such as multiple sclerosis (9–12).

During EBV infection, multifunctional viral glycoproteins bind to receptors and fuse with the cellular membrane, with the heterodimer gH-gL and the viral fusion protein gB being the core glycoproteins involved in this process (13). In this process, different receptor-bound glycoprotein complexes are required to mediate entry into various cell types. For example, gp350/CD21 interaction mediates its binding to B cells (14), while epithelial cell infection relies on specific receptors such as EphA2 (15) and NMHC-IIA (16). In recent years, significant progress has been made in receptor research, with R9AP being identified as having common receptor properties (17), providing new insights into the understanding of EBV infection mechanisms in different cell types.

EBV infection has two distinct characteristics: first, its host range is strictly limited, infecting only humans and other primates; second, it exhibits pronounced cell tropism, primarily targeting B cells and epithelial cells. These features are closely related to the distribution and recognition mechanisms of its receptors. A study has shown that EBV gp350 can effectively bind to the human receptor CD21 but lacks the ability to bind to mouse CD21, indicating a high species specificity of EBV. This binding specificity arises from the complementary structural and charge properties between gp350 and CD21 (18), which illustrates the important role receptors play in determining the virus’s selection of host cells. EBV exhibits limited cell tropism (13), which is closely related to the types of receptors. In B cells, it primarily relies on the binding of gp350/gp220 to CD21 (19), and membrane fusion is completed through the interaction of gp42 with human leukocyte antigen class II (HLA-II) (20). The gp42-HLA-II complex functions as a co-receptor, whose interaction mediates the activation of gH/gL/gB and induces membrane fusion, thereby enabling EBV entry into B cells (20). In contrast, although fibroblasts can bind to EBV to some extent, the virus cannot achieve effective infection due to the lack of key cofactors (21). This indicates that receptors and cofactors play a crucial role in determining the cell specificity of EBV infection.

Research on EBV receptors not only helps to reveal how the virus selects host cells but also provides potential targets for the development of vaccines and antiviral drugs. For example, vaccine design strategies centered around the gH/gL complex (22), or therapeutic approaches based on monoclonal antibodies or small molecule inhibitors (23, 24), etc., are expected to bring new breakthroughs in the prevention and treatment of EBV-related diseases. Meanwhile, the expression patterns of receptors could serve as potential biomarkers for risk stratification in high-risk populations and for the early screening of EBV-related tumors.

In this review, we systematically summarize the research progress on EBV receptors, exploring the mechanisms of action of different receptors and their significance in clinical applications. Additionally, we will discuss the potential research value of EBV receptors in disease treatment, vaccine development, and the prevention and control of infectious diseases, while offering new perspectives for the prevention and treatment of EBV-related diseases in clinical practice.

## CLASSIFICATION AND MECHANISMS OF EBV RECEPTORS

### B cell receptors

#### CD21

CD21 (CR2) is a ∼145 kDa membrane glycoprotein expressed on the surface of B cells, epithelial cells, and other cell types. It plays a role in bridging innate and adaptive immunity in the immune response (25). CD21 was the first EBV receptor to be discovered and characterized. In 1984, researchers initially treated B cells with monoclonal antibodies to block CD21 and observed that EBV was unable to bind to the cells. Then they subsequently transfected CD21 into *Staphylococcus aureus* particles, which then acquired the ability to bind EBV. These two sets of experiments together confirmed CD21 as the B cell receptor (BCR) for EBV (14).

CD21 relies on its short consensus repeat (SCR) domains to directly bind the viral envelope glycoprotein gp350/220, forming a CD21–gp350/220 complex that initiates viral entry (14, 19, 26–28). Cryo-electron microscopy and X-ray crystallography studies have demonstrated that the N-terminal domain of gp350 (particularly the D1/D2 regions) binds to the SCR1–SCD21 domains of CD21. This interaction involves electrostatic complementarity between acidic residues in CD21 (e.g., Glu37 and Asp82) and basic residues in gp350 (e.g., Lys/Lys/Arg clusters) (27–29). Each viral particle carries hundreds of gp350/220 molecules, allowing a single EBV virion to simultaneously engage multiple CD21 molecules. The clustering of CD21, in turn, causes the closely associated BCR to aggregate passively. BCR is the plasma membrane immunoglobulin on the B cell surface whose central function is to recognize and bind specific antigens, thereby initiating the B cell immune response. The associated Lyn/Syk tyrosine kinase signaling is activated and amplified, initiating a cascade of downstream pathways (30, 31). Degradation of IκBα activates the NF-κB pathway, in which NF-κB functions as a key transcription factor regulating inflammation and immunity, driving IL-6 gene transcription. The MAPK pathways (such as ERK1/2 and p38 kinases) and the JAK/STAT pathway are also activated, resulting in enhanced production of IL-6.

CD21 can bind complement C3d, and gp350 may pre-bind C3d to enhance infection efficiency through the C3d–CD21 interaction (27). Experimental evidence suggests that gp350–CD21 binding may induce membrane curvature changes that facilitate viral adsorption and endocytosis (32). Molecular dynamics simulations indicate that conformational changes in gp350 increase its binding affinity for CD21, while glycosylation may regulate CD21 recognition through steric effects (33). Deletion of the CD21 cytoplasmic domain does not impair viral binding but markedly reduces infection efficiency, suggesting a role in regulating endocytosis or lysosomal escape, while gp350 may facilitate viral proximity to the cell membrane through conformational changes and activate the gp42/gH/gL fusion complex to complete membrane fusion (28). Studies have shown that neutralizing antibodies targeting the EBV envelope glycoprotein gp350 can significantly influence the interaction between CD21/gp350 and then inhibit EBV infection in peripheral B lymphocytes (32, 34). Deletion of the intracellular domain of CD21 does not affect viral binding but markedly reduces infection efficiency, suggesting a potential role in regulating endocytosis or lysosomal escape. Furthermore, soluble recombinant CD21 can inhibit EBV infection in B cells *in vitro* (35), highlighting the essential role of this receptor in the viral entry process.

#### Human leukocyte antigen class II (HLA-II)

HLA molecules are cell surface glycoproteins whose primary function is antigen presentation (36). Some viruses (such as EBV) can utilize HLA-II molecules as entry receptors or co-receptors. However, this phenomenon is not widespread, as many viruses rely on their specific cell surface receptors for infection. HLA-II consists of an α chain and a β chain, each of which further folds into two functional domains: α1, α2, and β1, β2 (37). Following viral attachment, the viral glycoprotein gp42 binds to the β chain of HLA-II to trigger a membrane fusion signaling cascade. gp42 simultaneously engages gH/gL at one end and HLA-II at the other, forming the gH/gL–gp42–HLA-II complex, facilitating the fusion of the virus with the cell membrane and its invasion into the host cell (38–40). This process works in conjunction with the CD21 receptor, forming the classic mode of EBV infection in B cells. When exploited by viruses as a receptor, HLA-II not only confers specific cell tropism but also provides a mechanism for interfering with host immune responses. This allows the virus to establish latency within cells and evade immune clearance. Thus, HLA-II, when functioning as a viral receptor, not only influences host range and cell tropism but also creates favorable conditions for viral immune evasion and long-term persistence.

#### CD35

CD35 (CR1) can serve as an alternative receptor for EBV, mediating viral entry into certain B cells and epithelial cells, particularly those with low CD21 expression (29, 40). The interaction of gp350/220 with CD35 depends on its complement-binding domains SCR1–3. CD35 and CD21 may share partial binding epitopes, although CD35 exhibits lower binding affinity. CD35 can bind complement components such as C3b and C4b, and EBV may similarly exploit complement as a “bridge” to enhance infection. The cytoplasmic domain of CD35 differs from that of CD21 and may activate distinct downstream signaling pathways, such as anti-apoptotic pathways, thereby facilitating viral persistence (40).

### Epithelial cell receptors

#### Non-muscle myosin heavy chain IIA (NMHC-IIA) and neuropilin-1 (NRP1)

NMHC-IIA is a subtype of non-muscle myosin heavy chain, encoded by the Myh9 gene (41). It is involved in various physiological processes, including the maintenance of cell contractility, shape, and signal transduction (42). In 2015, Xiong et al. (16) discovered that the EBV glycoprotein gH/gL specifically binds to NMHC-IIA by means of immunoprecipitation and mass spectrometry analysis. Further experiments using siRNA knockdown and antibody blocking confirmed that NMHC-IIA is crucial for EBV infection of nasopharyngeal epithelial cells (NPECs). On the surface of susceptible spherical cells, NMHC-IIA aggregates extensively and co-localizes with viral particles, thereby greatly facilitating viral adhesion and entry.

Neuropilin-1 (NRP1) is a transmembrane glycoprotein consisting of 923 amino acid residues. It acts as a co-receptor for the vascular endothelial growth factor (VEGF) receptor tyrosine kinase in endothelial cells and plays an important role in VEGF-mediated endothelial functions (43). Additionally, NRP1 plays a critical role in the entry of various viruses, such as SARS-CoV-2 (44), hepatitis B virus (45), and enterovirus (46). In 2015, Wang et al. investigated the NRP1 receptor, showing that silencing the corresponding gene with miRNA or blocking expression using soluble NRP proteins significantly reduced the efficiency of EBV infection in nasopharyngeal epithelial cells (47). They further used confocal microscopy to observe colocalization of NRP1 with EBV at the cell membrane and demonstrated that NRP1 binds to EBV gB, thereby confirming that NRP1 mediates EBV entry into epithelial cells via this interaction.

NMHC-IIA and NRP1 are involved in the adhesion and internalization processes of EBV infection, respectively. NMHC-IIA cooperates with EphA2 to regulate cytoskeletal rearrangements and facilitate viral entry, possibly by inducing contraction of the actin network to form endocytic vesicles (48). NRP1 plays a facilitating role during endocytosis. As a co-receptor for VEGF, NRP1 forms complexes with VEGFR2 and TGF-β receptors. The CendR domain of EBV gB (a C-terminal arginine-rich motif) mimics the structure of natural ligands such as VEGF, enabling its binding to NRP1 and triggering macropinocytosis or lipid raft-dependent endocytosis to guide viral entry into cells (15). In addition, NRP1 engagement can activate intracellular signaling pathways, such as the EGFR–RAS–ERK cascade, potentially creating a more favorable intracellular environment for viral internalization and subsequent gene expression (47).

#### Ephrin receptor A2 (EphA2)

EphA2 is a member of the receptor tyrosine kinase Eph family, which constitutes the largest family of tyrosine kinase receptors. The extracellular domain of EphA2 contains conserved cysteine and fibronectin repeat sequences, mediating ligand binding, while the intracellular kinase domain transduces signals, potentially regulating development and inflammatory responses (49). EphA2 plays a role in the development of various organs in the human body (50–52). Additionally, it is a key regulator in tumorigenesis and cancer progression. Studies have shown that EphA2 is highly expressed in a variety of solid tumors, making it a preclinical target and a central focus in the development of anti-cancer therapeutic strategies (53). As early as 2012, EphA2 was identified as a receptor for the Kaposi’s sarcoma-associated herpesvirus, another human γ-herpesvirus (54). In 2018, Zeng’s and Longnecker’s group (15, 55) reported EphA2 as another important epithelial cell receptor. The researchers first employed bioinformatic analyses, comparing RNA-seq data sets of B cells and two commonly used EBV epithelial entry models (HEK293 and AGS cells), and identified EphA2 as the gene showing the greatest differential expression between these cell types. Then subsequent CRISPR–Cas9-mediated gene knockout experiments revealed a marked reduction in EBV fusion and infection efficiency, thereby validating EphA2 as a critical receptor for EBV infection of epithelial cells.

EphA2 interacts with the EBV gH/gL and gB glycoproteins to promote EBV internalization and membrane fusion. The viral glycoprotein directly binds to the fibronectin type III domain of EphA2, inducing receptor autophosphorylation on tyrosine residues and subsequently driving cytoskeletal rearrangements (56). EphA2 signaling regulates members of the Rho family of GTPases (including RhoA, Rac1, and Cdc42). Once activated, these GTPases orchestrate rapid actin polymerization and assembly beneath viral attachment sites, driving cytoskeletal remodeling and enabling EBV entry via clathrin-dependent endocytosis and membrane fusion (57). This interaction not only mediates viral entry but also activates the following signaling pathways (58): (i) PI3K/AKT pathway: Binding of EBV to EphA2 activates the PI3K/AKT signaling pathway, which is associated with cell survival, proliferation, metabolism, and inhibition of apoptosis. Sustained phosphorylation of AKT helps to create an intracellular environment favorable for viral latency. (ii) MAPK/JNK pathway: Phosphorylation of EphA2 at residue S901 activates the JNK signaling pathway, promoting the transition of EBV from latency to the lytic replication phase, during which the virus undergoes extensive replication and assembly. EphA2 possesses a dual identity as both an oncogene and a viral receptor. This unique property renders EphA2 a potential target for oncolytic virotherapy, enhancing viral selectivity toward tumor cells while simultaneously activating anti-tumor immunity (59). Moreover, this dual role provides a new theoretical basis for combined antiviral and anti-cancer therapeutic strategies.

#### Desmocollin 2 (DSC2)

Desmocollins (DSCs) are components of desmosomal proteins and consist of three isoforms, among which DSC2 is one (60). Dysregulation of DSC2 is associated with various diseases, while aberrant expression of DSC2 has been identified in various cancers, including gastric cancer (61), esophageal cancer (62), and prostate cancer (63). In a recent study in 2025 (64), researchers employed high-throughput approaches using recombinant EBV expressing green fluorescent protein (GFP) to conduct a genome-wide CRISPR–Cas9 screen, which identified DSC2 as a key factor in the HEK293 cell line. They subsequently performed a series of biochemical assays, then confirmed DSC2 as a critical receptor mediating EBV infection of epithelial cells. In another study published in the same year, using a similar approach, DSC2 was identified as an epithelial cell receptor for EBV, while DSC3 was characterized as an auxiliary factor facilitating infection. The study demonstrated that the combined application of antibodies against DSC2 and DSC3 effectively blocked cell-to-cell contact–mediated infection (65). Moreover, both studies showed that through its extracellular domain (preEC–EC2 region), DSC2 directly binds to the EBV gH/gL complex and enables viral entry independently of EphA2, and this interaction can be effectively inhibited by the corresponding antibodies, although the precise infection mechanism has not yet been fully elucidated (64, 65). To date, DSC2 has not been reported as a receptor for other viruses.

#### Integrins

Integrins are widely expressed on the surface of human cells and constitute a class of proteins that mediate cell–cell and cell–extracellular matrix recognition and adhesion. Previous studies have shown that many viruses exploit integrins to enter target cells, including adenoviruses, hantaviruses, reoviruses, and herpesviruses (66), and that such interactions can stimulate cellular endocytosis and activate infection-associated signaling pathways (67, 68). Several studies on EBV have also suggested that integrins play an important role in EBV adhesion to and invasion of epithelial cells. In 2007, a study reported that interactions between the EBV envelope protein BMRF2 and integrins α5β1 and α3 contribute to the infection of polarized epithelial cells (69). Fletcher et al. (70) demonstrated through a series of cellular and biochemical assays that integrins αvβ6 and αvβ8 serve as specific receptors for the viral glycoprotein gH/gL, and that their interaction triggers EBV adhesion to and fusion with epithelial cells. αvβ6/αvβ8 may promote EBV infection either by activating indirect signaling pathways, such as the FAK/Src pathway, leading to Rho GTPase-mediated cytoskeletal rearrangements, or by modulating the cytoskeleton through direct interactions with gH/gL. In addition, their study reported that integrins act as favorable triggers for EBV infection of epithelial cells rather than B cells, which may be associated with the viral tropism (71). The mechanisms of the EBV receptors studied to date are summarized in Table 1.

**TABLE 1:** Mechanisms of different EBV receptors

Receptor name	Cell type	Viral envelope glycoprotein	Primary function	Downstream signaling pathways and effects	References
CD21	B cell	gp350	Mediate EBV attachment	CD21 activates BCR-associated Lyn/Syk tyrosine kinase signaling to promote viral internalization	14, 32, 33
HLA-II	B cell	gp42	Mediate anchoring and trigger membrane fusion	HLA-II triggers gB to execute membrane fusion and may indirectly regulate immune signaling	27, 38, 40
Integrins (αvβ6/αvβ8)	Epithelial cell	gH/gL	Participate in viral adhesion and membrane fusion	Integrins αvβ6/αvβ8 activate the FAK/Src signaling pathway and facilitate viral endocytosis	70, 71
NMHC-IIA	Epithelial cell	gH/gL	Provide mechanical force and facilitate anchoring	NMHC-IIA interacts with actin and coordinates membrane remodeling through Rho GTPases	16, 48
NRP1	Epithelial cell	gB	Assist membrane fusion	NRP1 modulates growth factor signaling pathways, such as VEGF and TGF-β, and assists in membrane fusion	47
EphA2	Epithelial cell	gH/gL	Facilitate fusion and internalization	EphA2 induces receptor dimerization and phosphorylation, activates the PI3K/Akt pathway, and triggers membrane fusion	15, 55, 58
R9AP	B cell/epithelial cell	Multiple glycoprotein	Mediate EBV membrane fusion with B cells and epithelial cells	R9AP initiates gH/gL-gB mediated membrane fusion, which may involve G protein signaling regulation	17
DSC2	Epithelial cell	gH/gL	Mediate fusion and internalization without EphA2	DSC2 mediates membrane fusion and internalization independently of EphA2, with the specific pathway yet to be elucidated	64, 65

### Common receptor R9AP

R9AP was initially identified through its interaction with RGS9-1 in detergent extracts of rod outer segment membranes. It is predominantly expressed in the retina, where it binds to the N-terminal domain of RGS9-1 and anchors it to disc membranes via its C-terminal transmembrane helix (72, 73). In 2025, the discovery of the common receptor R9AP has opened a new phase in EBV receptor research (17). Researchers compared sphere-like cells, which display high EBV infection efficiency, with monolayer cells, which are less susceptible, using genome-wide microarray analysis to identify differentially expressed genes. siRNA-mediated silencing of transcripts more highly expressed in sphere-like cells revealed that knockdown of R9AP dramatically reduced EBV infection efficiency. Conversely, R9AP overexpression enhanced EBV infection efficiency by approximately threefold compared with empty vector controls. They further performed antibody-blocking and immunoblotting assays, confirming that R9AP plays a critical role in EBV infection of both B cells and epithelial cells. The N-terminal domain of R9AP can be exposed on the cell surface, a critical feature that enables direct and specific binding to the EBV gH/gL glycoprotein complex. This interaction triggers gH/gL–gB-mediated membrane fusion, and such bidirectional localization underscores the pivotal role of R9AP in the shared infection mechanisms of both B cells and epithelial cells. In addition, the study found that highly competitive neutralizing antibodies, such as AMMO1, are able to block the interaction between R9AP and the gH/gL complex (17).

**Fig 1:**
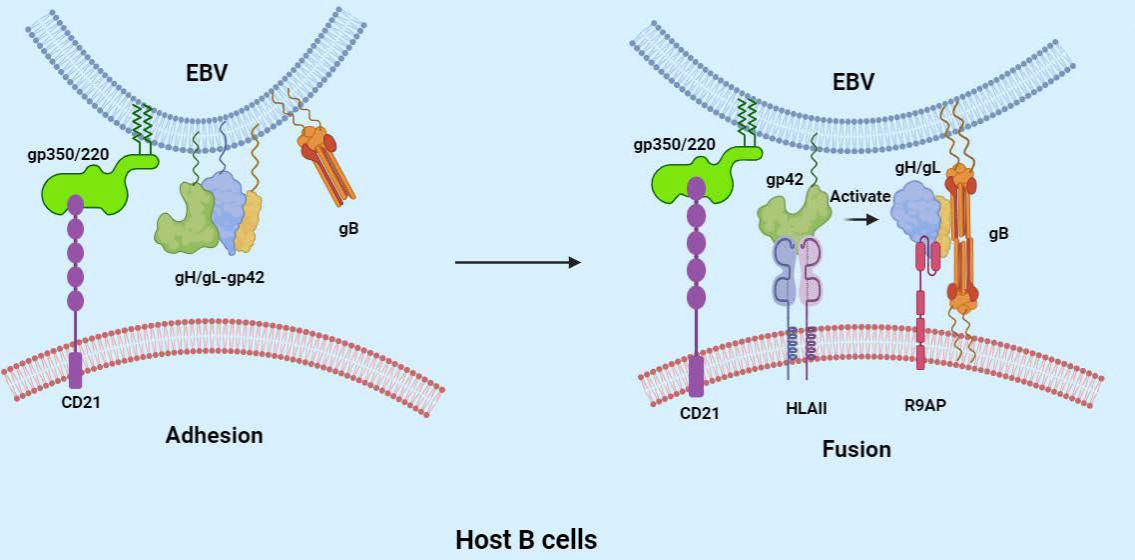
Mechanism of EBV infection in B cells. In B cells, gp350 binds to CD21 to mediate EBV adhesion. Subsequently, gp42 interacts with HLA-II and undergoes conformational changes, which activate gH/gL binding to R9AP and ultimately trigger membrane fusion between the virus and the host cell via the gH/gL–gB complex.

**Fig 2:**
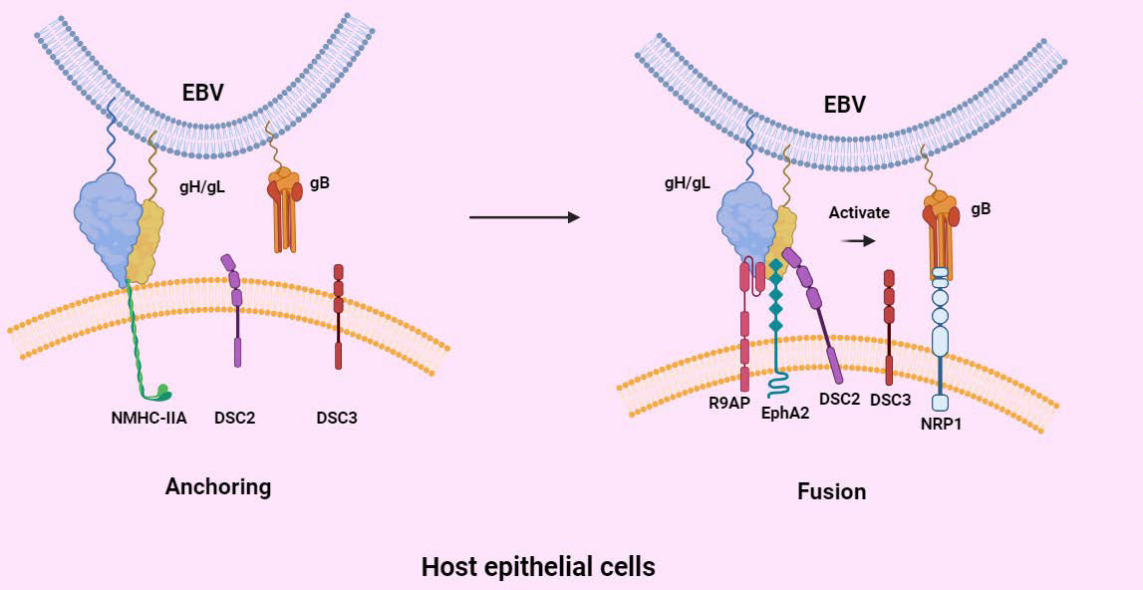
Mechanism of EBV infection in epithelial cells. In epithelial cells, NMHC-IIA interacts with gH/gL to mediate EBV anchoring. Concurrently, gH/gL binds to both R9AP and EphA2, and gB binds to NRP1, collectively activating gB-mediated membrane fusion between the virus and the host cell. In addition, DSC2 plays a critical role in both anchoring and membrane fusion, while DSC3 is characterized as an auxiliary factor facilitating infection.

R9AP does not act in isolation but coordinates with other receptors; it coordinates with the EBV gp42–HLA-II complex in B cells and the gH/gL–EphA2 complex in epithelial cells to mediate viral and cellular membrane fusion. Previous studies have demonstrated that gH/gL and gB are essential glycoproteins for EBV infection of all host cells. The complex formed by gH/gL and host cell surface receptors plays a critical role in subsequently activating gB to mediate membrane fusion and viral internalization. The receptor-mediated mechanisms by which EBV infects B cells and epithelial cells are illustrated in Fig. 1 and 2, respectively.

## PROSPECTS FOR THE APPLICATION OF EBV RECEPTORS

### Vaccine development

EBV can induce a variety of diseases, yet no EBV-specific therapeutics are currently available. Therefore, preventive vaccines represent the most effective and cost-efficient method for controlling EBV-related diseases. Given that EBV is increasingly recognized as a triggering factor for autoimmune diseases, the development of EBV vaccines has become even more important (74, 75). The fundamental principle of vaccine development is to utilize the antigen–antibody reaction to prevent EBV infection of target cells such as B cells and epithelial cells. Although no EBV vaccine has been licensed to date, significant progress has been made, with several candidates currently undergoing clinical evaluation, including vaccines based on viral surface proteins, viral vector-based vaccines, and mRNA vaccines.

gp350, gH, gL, gB, and gp42 are key membrane proteins involved in EBV infection of B cells, whereas membrane proteins such as gH, gL, gB, and BMRF2 participate in EBV infection of epithelial cells (39, 76–78). These EBV envelope proteins are components of the core viral fusion mechanism and may serve as promising targets for preventive EBV vaccines.

gp350 is the most abundant EBV envelope protein, accounting for approximately half of the neutralizing activity in EBV-positive human sera (76). Consequently, early vaccine development efforts focused primarily on gp350. Owing to the limited efficacy of the monovalent gp350 vaccine, researchers further investigated and developed gp350-based nanoparticle vaccine. An EBV gp350–ferritin nanoparticle, designed using epitope peptides from the receptor-binding domain of gp350, elicited potent neutralizing antibodies and showed considerable promise in both protective efficacy and antibody durability (79). A large-scale clinical trial with soluble recombinant gp350 demonstrated reduced incidence of infectious mononucleosis (IM) following EBV infection (80). However, the small sample size of this trial may limit its statistical significance, and further studies are required to strengthen the reliability of the findings. EBV gH/gL or gH/gL/gp42 nanoparticle vaccines can effectively inhibit EBV infection of both B cells and epithelial cells, with stronger efficacy than the corresponding soluble glycoproteins (76). Other studies reported that when EBV gH/gL or gH/gL/gp42 nanoparticle vaccines were combined with a gp350 nanoparticle vaccine [gp350D(123)], no immune competition was observed (81), making combined vaccines of gH/gL/gp42–ferritin and gp350-based vaccines attractive candidates.

With the increasing depth of research on EBV receptors, the development of vaccines targeting the interactions between EBV and its receptors has emerged as a promising direction. The recently identified common receptor R9AP represents a potential novel target for the prevention of EBV infection (17), and vaccines could be designed to target the R9AP–gH/gL interaction interface based on its mechanism of action, which may elicit antibodies capable of conferring protection against EBV infection of both B lymphocytes and epithelial cells, thereby enhancing vaccine efficacy. The newly identified DSC2 enables cells normally not susceptible to EBV to become infectable, working in synergy with EphA2 during EBV infection (64). Blocking the interaction between gH/gL and DSC2 may therefore offer an additional potential target for EBV vaccine development.

With the approval by the US Food and Drug Administration (FDA) of the mRNA-based Moderna vaccine for the prevention of COVID-19 (82), together with rapid advances in mRNA vaccine research for viral infection prevention and cancer therapy, mRNA vaccines targeting EBV have emerged as a major focus of investigation. Recent studies have shown that mRNA-based therapeutic vaccines targeting domains of EBV latent proteins enriched in T-cell epitopes can effectively induce both cellular and humoral immune responses, thereby suppressing tumor progression (83). In addition, the mRNA-based therapeutic cancer vaccine WGc-043 has received dual IND approvals from the National Medical Products Administration (NMPA) of China and FDA, highlighting mRNA vaccines as providing additional therapeutic options for EBV-associated malignancies.

### Antiviral drug development

EBV is the first human oncogenic virus proven to cause cancer and is closely associated with infectious mononucleosis, various lymphomas, and epithelial malignancies, particularly nasopharyngeal carcinoma (84). Antiviral drug development for EBV generally follows two strategies: (i) designing agents targeting EBV nucleic acids or core proteins to block infection, and (ii) developing antibodies against viral receptors to prevent EBV binding to B cells and/or epithelial cells. Here, we focus on receptor-targeting antibody development, including receptors such as CD21, EphA2, and R9AP (85).

The specific interaction between CD21 and the EBV surface glycoprotein gp350 provides an important target for antiviral drug development (32). Joyce et al. (28) structurally defined the CD21-binding site (CD21bs) on gp350 and revealed structural similarities between this site and epitopes recognized by several rhesus and human monoclonal antibodies. These findings indicate that the CD21bs on gp350 represents a highly neutralization-sensitive antigenic supersite and a promising therapeutic target.

The development of EphA2-targeted therapeutics has also emerged as an active area of research, as such agents can effectively inhibit tumor growth and restore drug sensitivity in resistant tumor cells (15, 55). Zeng et al. (15) reported that blockade of the EphA2 extracellular domain impaired EBV infection of epithelial cells, and that EphA2 antibodies, EphrinA1, and the inhibitor 2,5-dimethylpyrrole benzoic acid successfully blocked viral entry. More recently, Yang et al. (86) discovered that interferon-induced transmembrane protein 1 (IFITM1) exerts an opposing effect to EphA2 in EBV infection: IFITM1 competitively blocks EphA2 binding to EBV glycoproteins, thereby inhibiting viral entry into epithelial cells, whereas YTHDF3 (an m6A reader) suppresses IFITM1 expression via degradation of DEAD-box protein 5 (DDX5). Based on the mechanism of IFITM1, either by modulating IFITM1 expression or exploiting its competitive interaction with EphA2 may provide a novel therapeutic avenue to reduce EBV-associated disease risk. Collectively, these findings highlight EphA2 as a promising therapeutic target to block EBV infection of epithelial cells.

The discovery of the common receptor R9AP provides a new research direction for the treatment of EBV-associated diseases. A monoclonal antibody targeting R9AP (5E9) can inhibit EBV infection of human primary B cells and primary nasopharyngeal epithelial cells (NPECs), demonstrating the feasibility of anti-R9AP antibodies for combating EBV infection (17). Because the N-terminal 50 residues of R9AP are critical for supporting EBV infection, the team added exogenous peptides at the N-terminus and found that R9AP^19–30^ conferred partial protection against EBV infection. Targeting R9AP may thus represent a potential strategy against EBV infection, providing new perspectives for antiviral drug development. Designing blockade strategies against R9AP could simultaneously inhibit EBV infection of B cells and epithelial cells, significantly enhancing drug efficacy. The unique structure and interaction mechanisms of R9AP also provide valuable targets for future development of more specific and broad-spectrum antiviral drugs.

DSC2 acts as a key upstream receptor that cooperates with EphA2 to mediate EBV infection of epithelial cells, providing a basis for the development of novel antiviral therapeutics. The extracellular domain of DSC2 that interacts with the EBV gH/gL complex can be targeted by polyclonal antibodies, thereby blocking EBV infection of primary epithelial cells (64). The identification of DSC2 offers a potential molecular target for precision therapies against EBV-related diseases, as antibodies designed to target its extracellular binding domain can directly inhibit EBV infection of epithelial cells.

### Early screening and diagnosis

EBV contributes to the development of nasopharyngeal carcinoma and other malignancies, making early screening and diagnosis of EBV-associated diseases critically important. The identification and application of EBV-specific biomarkers is therefore a major research focus. As novel EBV receptors continue to be identified, receptor-based early screening technologies are also being developed. Li Xin and colleagues observed a potential inverse correlation between IFITM1 expression and EBV infection in nasopharyngeal carcinoma tissue samples and EBV-positive/negative cell lines. They showed that IFITM1 competitively blocks EphA2 binding to EBV glycoproteins gH/gL and gB (86). The association between EBV infection and IFITM1 levels provides a novel perspective for early screening, suggesting IFITM1 as a potential biomarker for EBV-related diseases. The recently identified host-determining receptor DSC2 renders non-susceptible cells permissive to EBV infection, and its expression is strongly correlated with viral infectivity (64). Thus, DSC2 holds promise as a biomarker to accelerate research and clinical application in the early diagnosis of EBV-related diseases such as nasopharyngeal carcinoma. With the continuous advancement of research, EBV receptors hold promise for providing new approaches and methods for the early screening and diagnosis of EBV-related diseases in clinical practice.

## OPEN QUESTIONS

In recent years, research and experimental studies on EBV have advanced significantly worldwide, yielding important findings. With the discovery of the common receptor R9AP, scientists have gained a more comprehensive understanding of the diverse mechanisms by which EBV infects host cells. However, the receptors identified so far are restricted to the surfaces of human B cells and epithelial cells, raising the question of whether additional, yet unidentified receptors exist. Can EBV infect and cause disease through other cell types, such as neurons or T cells? These are critical questions driving further progress in this research field.

Compared with B cells and epithelial cells, relatively few studies have reported a tropism of EBV for T cells. In 1988, a study detected EBV in tumor cells derived from patients with T-cell lymphoma (87), and subsequent studies have similarly reported the presence of EBV-infected T cells in certain T-cell disorders, including T-cell lymphomas and T-cell lymphoproliferative diseases (88–90), suggesting an important role for EBV infection in the pathogenesis of T-cell-associated diseases. In 2020, a study by Nicolas et al. (91) reported that CD21 is a required receptor for EBV infection of T cells. The study further demonstrated that, in contrast to the predominant strain EBV type 1 (EBV-1), the less common EBV type 2 strain (EBV-2) is capable of infecting primary T cells both *in vitro* and *in vivo*. The authors subsequently performed a series of antibody neutralization experiments, which showed that EBV infection of CD3^+^ T cells was neutralized by antibodies against the viral glycoprotein gp350 as well as by antibodies targeting cellular CD21, thereby confirming that the gp350 glycoprotein and the CD21 receptor are essential for this type of T-cell infection. At present, several questions regarding EBV infection of T cells remain under investigation, including differences in CD21 expression among distinct T-cell subsets and the reasons why EBV-2, but not EBV-1, is capable of infecting T cells. However, this work not only provides new insights into the clinical treatment of T-cell lymphomas and the development of related vaccines but also suggests the existence of additional, as-yet-unidentified receptors on T cells.

Multiple clinical studies have confirmed that EBV is associated with various neurological disorders, including primary central nervous system lymphoma, multiple sclerosis, Alzheimer’s disease, cerebellar ataxia, and Parkinson’s disease (92–96). Elevated EBV titers have been detected in the cerebrospinal fluid of affected patients (97, 98). However, the prevailing view has long held that EBV primarily infects B cells and epithelial cells, without directly affecting neurons (1, 99, 100). In 2015, a study provided the first evidence that EBV can infect human primary neurons (101). After exposing three distinct neuronal cell lines to GFP-tagged EBV strains for a period of time, researchers observed green fluorescence signals distributed across various regions of each neuronal cell. They further measured the levels of viral-encoded antigens within the neuronal cells, finding significant increases in EBNA1, gp350, and BZLF1 expression. These results indicated that EBV had successfully infected the neurons and undergone lytic replication to produce progeny virions. In addition, the experiments suggested that the antiviral drug acyclovir effectively inhibited viral lysis and replication within infected neurons, offering a potential therapeutic avenue for EBV-associated neurological diseases. Another study reported that EBV can infect astrocytic cell lines such as U-87MG *in vitro*, with significantly increased expression of the latency-associated proteins LMP1 and EBNA1.

In addition to directly infecting neurons, EBV can also affect the nervous system through indirect pathways. For example, a recent study established a humanized mouse model and demonstrated that EBV infection induces the expansion of T-bet^+^ CXCR3^+^ B cells. These B cells migrate to specific regions beneath the meninges, where they attract T cells, thereby contributing to the pathogenesis of multiple sclerosis (74). Another study investigating EBV immune evasion reported that, in the brain tissue of multiple sclerosis patients, EBV-infected B cells exhibit high expression of PD-L1. These cells engage with infiltrating PD-1^+^ CD8^+^ T cells, thereby suppressing the immune microenvironment and promoting persistent EBV infection as well as disease progression (102). Moreover, although EBV has long been considered capable of infecting only human cells (103), a recent study demonstrated that ectopic expression of human DSC2 in an EBV-insensitive hamster cell line rendered these cells permissive to EBV infection (64). This breakthrough helps overcome the longstanding barrier posed by the lack of EBV infection models in animals, providing critical technical support for future studies of EBV-associated diseases and the development of novel therapeutics using animal models.

At present, the receptors and precise mechanisms underlying EBV infection of non-B and non-epithelial cells remain poorly defined. Nevertheless, multiple studies have expanded our understanding of EBV neurotropism, showing that EBV not only infects the host via B-cell or epithelial cell receptors but can also modulate the microenvironment through diverse pathways, thereby contributing to disease pathogenesis. With the rapid development of high-throughput sequencing, genomics, and relevant animal models, research on receptors in non-B and non-epithelial cell types will continue to advance and is expected to become a central focus in this field.

## CONCLUSION

In the study of human EBV and its associated diseases, research on EBV receptors plays an indispensable role. This review summarizes all currently known EBV receptor types, compares their functions and the specific mechanisms by which they mediate viral infection, and discusses future research directions as well as potential applications of these receptors. In the discussion section, we also highlight unresolved key questions and propose new perspectives, aiming to provide insights and approaches for future research in this field. Although notable progress has been made in EBV receptor research, the mechanisms of action for some receptors remain incompletely understood. Future studies should focus on the identification of novel receptors and the elucidation of their underlying mechanisms, thereby providing a theoretical foundation for the development of EBV vaccines and targeted therapeutics in clinical practice.
